# Toxicity study of UV/ZnO treated solution containing Reactive blue 29 using *Daphnia magna* as a biological indicator

**DOI:** 10.1016/j.mex.2019.03.019

**Published:** 2019-03-28

**Authors:** Mohammad Hadi Dehghani, Masoumeh Mahmoodi, Ahmad Zarei

**Affiliations:** a Department of Environmental Health Engineering, School of Public Health, Tehran University of Medical Sciences, Tehran, Iran; b Institute for Environmental Research, Center for Solid Waste Research, Tehran University of Medical Sciences, Tehran, Iran; c Department of Environmental Health Engineering, Faculty of Health, Social Development and Health Promotion Research Center, Gonabad University of Medical Sciences, Gonabad, Iran

**Keywords:** Biological test, *Daphnia magna*, Toxicity, Reactive blue 29 dye, UV/ZnO, Nanophotocatalysis process

## Abstract

Effluents from textile industry are a major source of environmental pollution, especially water pollution. One of the commonly used nanoparticles is zinc oxide (ZnO). In this study, the toxicity of solutions containing the Reactive blue 29 dye after nano-catalytic process UV/ZnO using the model organism *Daphnia magna* (*D. magna*) was studied. This is a fundamental – practical study, which was done at laboratory scale. *D. magna* is considered as an indicator of textile industry wastewater’s toxicity. First, biological tests were performed according to the Standard Methods for the Examination of Water and Wastewater. Results showed that the LC_50_ values at 24, 48, 72, and 96 h were 80.27, 62.26, 49.42, 32.64 mg/L, respectively.

Advantages of this technique includes:

• The results of this study can be used for selection of the appropriate methods for the removal of the dyes from wastewater.
• Possible hazard of toxicity exists to ecosystems in any receiving system, highlighting the need for evaluating the toxicity of industrial dyes.
• The results of this study show that *Daphnia magna* is a suitable indicator for evaluating the toxicity of contacted oxidizing nanoparticles with a Reactive blue 29 dye.

## Graphical abstract

**Figure:**
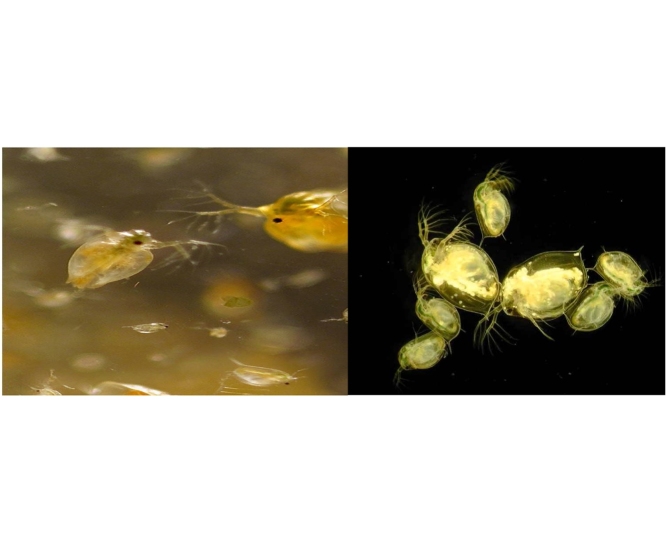

**Specifications Table**

**Table:** 

Subject area:	Environmental sciences
More specific subject area:	Toxicity assessment, nanoparticles, dye
Protocol name:	Toxicity study of UV/ZnO treated solution containing Reactive blue 29 using *Daphnia magna* as a biological indicator
Reagents/tools:	The toxicity of the solution containing Reactive blue 29 dye after Nano-catalytic process UV/ZnO using biological test *Daphnia magna* (*D. magna*) was studied
Experimental design:	The data of effects of main experimental parameters were acquired.
Trial registration:	No applicable
Ethics:	No applicable

## Description of protocol

Rapid population growth, urbanization and industrialization have led to the pollution of many surface and groundwater resources globally [[1], [2], [3], [4], [5], [6], [7]]. Among water pollutants, dyes are considered as an important group used in many industries including textile dyeing, paper printing and other sectors such as photography, pharmaceuticals, food and cosmetic industries. Effluents from textile industry are a major source of environmental pollution, especially water pollution due to highly colored species and toxicity to some organisms. Here *Daphnia magna* is used as a biological indicator for the assessment of toxicity.

## Materials

**Fig. 1:**
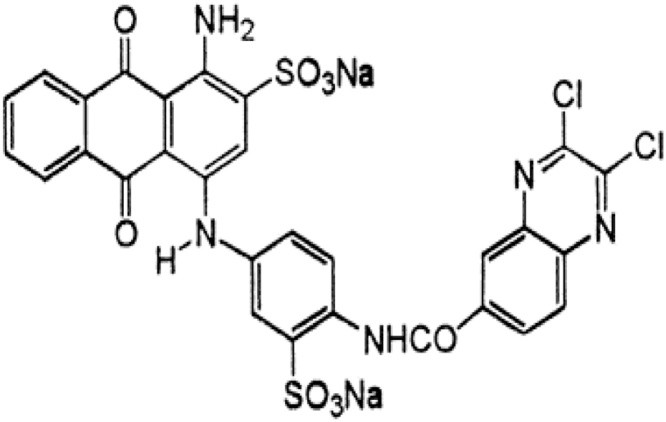
Structural formula of Reactive blue 29 [11].

Reactive blue 29 dye is an anionic and water-soluble dye. The chemical formula of this dye is C_29_H_15_Cl_2_N_5_Na_2_O_9_S_2_ with a molecular weight of 786 and maximum absorption wavelengths of 596, 597, and 600 nm [[8], [9], [10], [11]]. Its chemical formula is presented in Fig. 1.

## Procedure

Zinc oxide nanoparticles (ZnO), a white to yellow color powder with a hexagonal crystalline structure and a specific surface area of 40–150 m^2^/g and a bulk density of 150 kg/m^3^, purity of 99.8 and grain size 6–12 nm was used as an adsorbent in this study. The most important stage for bio testing with *D. magna* is the infancy stage, at which their neonates’ size reaches 0.8–1 mm [12,13]. The reason of using *Daphnia* neonates instead of adult *Daphnia* is that if adults are used, reproduction and increase of the number of *Daphnia* in the samples could be possible, which causes interference in the results of the experiments and uncertainties. To perform the toxicity experiments, after preparing the adsorbent dosages (0.1, 0.2, and 0.3 mg/L), a certain amount of the adsorbent was added to Reactive blue 29 dye stock solution with the initial concentration of 100 mg/L. Thereafter, this solution was mixed on a magnetic stirrer for 20 min in a dark place. After that, the solution was transferred to ultraviolet reactor to be exposed to ultraviolet radiation with a constant mixing to prevent any nanoparticles sedimentation. In these experiments, Phillips HPLN 125 W (${\text{λ}}_{\text{u}\text{v}}\;\geq \;254\;\text{n}\text{m}$) lamp was used as the light source. To avoid the radiation effects on human, the reactor was covered with aluminum foil [14]. After the predetermined duration, the toxicity experiments were performed. Daphnia is extensively used as a test organism in aqueous toxicology due to their small size, short life cycle, amenability to laboratory culture and a high sensitivity to toxicants [15]. The laboratory *D. magna* culture were obtained from the microbiology laboratory of Environmental Health Engineering at Faculty of Health at Tehran University of Medical Sciences. Upon arrival at our laboratory, the *D. magna* were gradually acclimated to synthetic freshwater in an incubator. The synthetic water freshwater water for *D. magna* was hard water (MgSO_4_ 246 mg/L, NaHCO_3_ 192 mg/L, CaSO_4_ 120 mg/L and KCl 8 mg/L), with a hardness of 168 mg CaCO_3_/L, alkalinity 115 mg CaCO_3_/L, a dissolved oxygen concentration >5 mg/L, and pH 7.5. This water was then left to stand for at least two weeks prior to use in the experiments. During the acclimation period, as also during experiments, *D. magna* were fed algae. The *Daphnia* culture medium was replaced on a weekly basis and aerated using an aquarium air pump mildly. Measurements were made to ensure that water remained sufficiently oxygenated (with ninety percent saturation) and the solution pH and temperature were monitored during the test period [16]. For the experiments, *Daphnia* neonates were used, and during the culture duration, the temperature was constant at around 25 °C. Neonates (lower than 24 h) were collected. *Daphnia* neonates (lower than 24 h) were collected for experiments using a plastic pipette and washed by diluted water for 5 min three times. *D. magna* were fed phytoplankton and a slurry of yeast, Cerophyll and trout chow (18.5 mg dry weighty per liter). Then, they were moved to the exposure chamber and exposed to the dye. Thereafter, a series of beakers was chosen, and one of them was considered as the control, in which the concentration of the material under investigation was zero. All beakers used were new and were acid washed in 1% Nitric acid and rinsed completely with synthetic water prior to use. Ten *Daphnia* were added to each experimental beaker. In the next stage, 5, 10, 20, 30, 40, 50, 75, and 100 mg/L dye were prepared and observations were made after 24, 48, 72, and 96 h. At the end of the test, the number of live neonates were recorded. Each test was carried out in duplicates. Immobility or mortality was checked at 24 and 48 h and organisms that were unable to swim within 15 s after gentle shaking were considered dead [17]. Based on the international and national guidelines regarding *Daphnia magna* assay, the percentage mortality or immobilization of the organisms in the control system should be ≤10% at the end of the exposure period. The toxicity experiments was investigated by the value of LC50, a concentration of the compounds causing death to 50% of *Daphnia* during incubation with toxic matter.

## Toxicity assessment

**Fig. 2:**
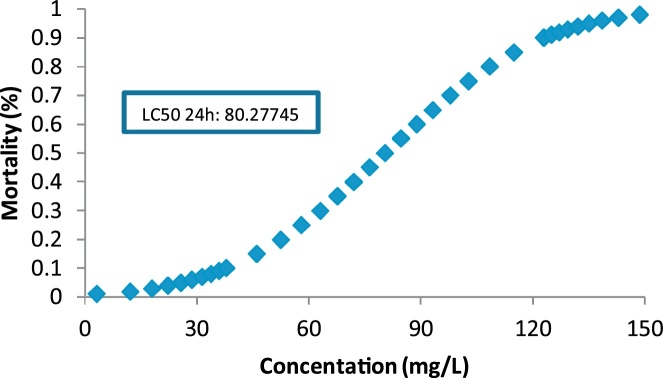
LC50 determination for 24 h exposure.

**Fig. 3:**
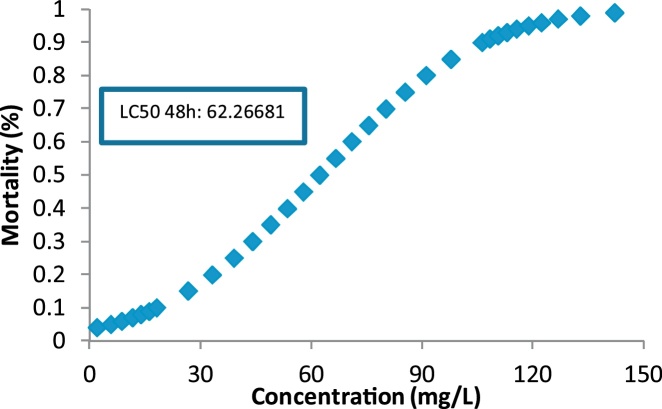
LC50 determination for 48 h exposure.

**Fig. 4:**
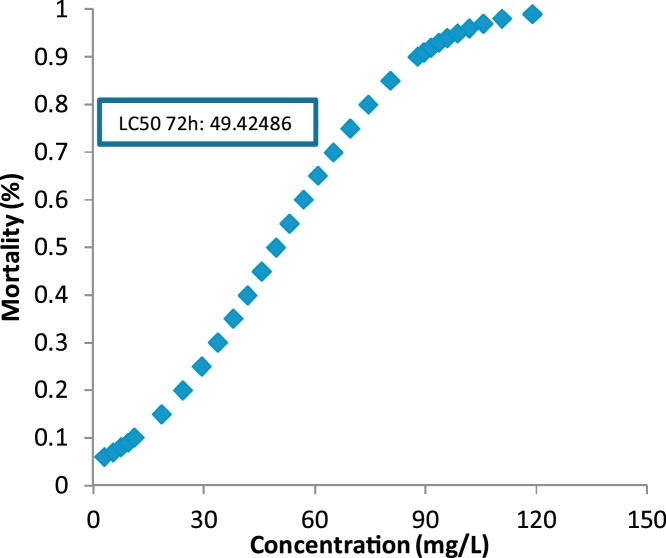
LC50 determination for 72 h exposure.

**Fig. 5:**
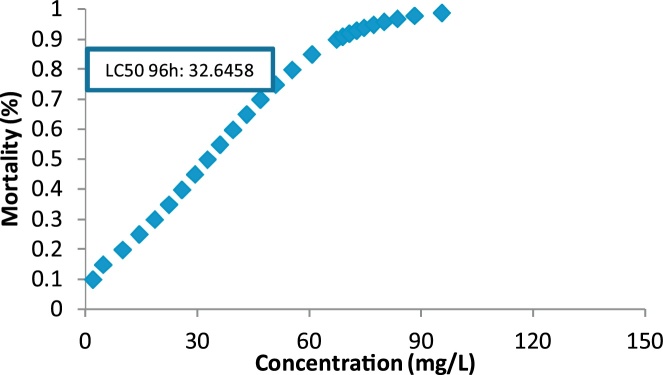
LC50 determination for 96 h exposure.

The results of toxicity assessment in 24, 48, 72 and 96 h are given in Table 1. Based on the table, higher exposure time resulted in higher death number of neonates. The results of LC50 determination are also given in Fig. 2, Fig. 3, Fig. 4, Fig. 5.

**Table 1:** Data of Reactive blue 29 toxicity on *D. magna* using samples of UV/ZnO nanophotocatalytic reactor.

Concentration of dye (mg/L)	Number of *D. magna* tested	Number of *D. magna* death during the experiments (Number per hour)			
		24 (h)	48 (h)	72 (h)	96 (h)
100	10	7	8	10	10
75	10	4	6	6	8
50	10	2	4	6	8
40	10	2	4	4	7
30	10	1	3	4	6
20	10	0	1	2	4
10	10	0	0	1	3
5	10	0	0	0	0
0	10	0	0	0	0

## Conflict of interest

The authors of this article declare that they have no conflict of interests.
